# Fetal exposure to famine increases the risk of cardiovascular disease in adulthood: findings from a population-based screening study

**DOI:** 10.3389/fcvm.2025.1473602

**Published:** 2025-07-28

**Authors:** Hui Li, Minjie Qi, Shuxian Yang, Hanxue Zhang, Liang Chang, Yan Gao, Lei Fan, Kai Kang

**Affiliations:** Institute for Prevention and Control of Chronic Non-communicable Diseases, Henan Provincial Center for Disease Control and Prevention, Zhengzhou, Henan, China

**Keywords:** famine, early life, CVD, Henan, China PEACE

## Abstract

**Introduction:**

Undernutrition during early life may increase the risk of chronic diseases in adulthood. The study aimed to investigate whether fetal exposure to famine would increase the risk of cardiovascular disease (CVD) in adulthood.

**Materials and methods:**

Data were collected from 16 sites in Henan by the China Patient-centered Evaluative Assessment of Cardiac Events (PEACE) Million Persons Project. The famine-exposed group was defined as participants born between 1 January 1959 and 31 December 1961, and the non-exposed group was defined as participants born between 1 January 1955 and 31 December 1957, and those born between 1 January 1963 and 31 December 1965. Multivariate logistic regression models were used to explore the association between fetal exposure to famine and CVD in adulthood, with adjustments for age, sex, education, family’s annual income, currently smoking, drinking alcohol, body mass index, anti-hypertensive drugs, anti-diabetic drugs, and lipid-lowering drugs.

**Results:**

Fetal exposure to famine increased the risk of total CVD [odds ratio (OR) = 1.25, 95% confidence interval (CI): 1.14–1.38], coronary heart disease (OR = 11.25, 95% CI: 1.05–1.47), stroke (OR = 11.22, 95% CI: 1.09–1.36), and 10-year CVD risk (OR = 11.22, 95% CI: 1.14–1.31) compared with the non-exposed group. The stratified analysis suggested that after being exposed to famine in the fetal period, men had a higher risk of CVD than women in adulthood (men: OR = 11.26, 95% CI: 110–1.44; women: OR = 11.23, 95% CI: 1.12–1.35) and the population in rural areas had a higher risk of CVD than that in urban areas (rural: OR = 11.30, 95% CI: 1.15–1.48; urban: OR = 11.20, 95% CI: 1.05–1.39).

**Discussion:**

Fetal exposure to famine increased the risk of total CVD, coronary heart disease, stroke, and 10-year CVD risk in Henan. The association was more pronounced in men and rural areas.

## 1 Introduction

Cardiovascular disease (CVD), principally referring to ischemic heart disease (IHD) and stroke, has surpassed infectious disease to become the primary cause of mortality worldwide, with high morbidity in recent decades (1). In 2019, CVD became the leading cause of death among the four major chronic non-communicable diseases (CVD, cancer, chronic respiratory, and diabetes) globally, resulting in a total of 17.9 million deaths (2). The prevalence and disease burden of CVD are rising in China. It was estimated that, in total, there were 94 million individuals with CVD in 2016, resulting in 3.97 million deaths and 78.11 million disability-adjusted life years (DALYs) (3). The Report on Cardiovascular Health and Diseases in China 2021 suggested that the total hospitalization cost of inpatients with CVD had risen in 2019 in comparison with 2016, amounting to 1,773.38 billion RMB (1 RMB = 0.15 USD) (4). Rapid economic development in Henan has been accompanied by urbanization, lifestyle transformation, and population aging. Moreover, CVD is a significant public health issue. In 2017, the number of deaths and DALYs attributed to CVD were 375,000 and 7.3 million, respectively (5). Given this, the huge disease and economic burdens caused by CVD deserve attention, and it is imperative to implement strategies and measures to prevent and control CVD. Unhealthy lifestyle habits, including smoking, drinking alcohol, eating an unbalanced diet, and a lack of physical exercise, are commonly regarded as risk factors for CVD (4). Furthermore, the fetal origin theory proposes that undernutrition in the fetal or infant stage increases the risk of chronic non-communicable disease, including obesity, diabetes, hypertension, and CVD, in adulthood (6–8). However, exposing an infant to malnourishment during early life is unethical in studies on human beings, and, therefore, famines have been widely used as an alternative tool to confirm the hypothesis. The Great Chinese Famine is recorded as a “three-year natural disaster” in Chinese literature, typically from 1959 to 1961, and resulted in excessive death and loss of fertility due to food shortages (9). While the relationship between exposure to famine in early life and CVD in adulthood has been explored in other provinces, such as Guangdong (10), few studies have been conducted in Henan, an agricultural and populous province, which experienced a severe famine. Therefore, the data from a cross-sectional study in Henan conducted by the China Patient-centered Evaluative Assessment of Cardiac Events (PEACE) Million Persons Project was used to examine whether exposure to famine in the fetal period increases the risk of CVD in adulthood.

## 2 Subjects and methods

### 2.1 Study design and subjects

This study was based on the China PEACE Million Persons Project. The project was a nationally representative study directly funded by the government, covering all 31 provinces, autonomous regions, and municipalities, that screened individuals with high CVD risk and has been described in detail previously (11). In total, 16 sites across Henan were selected and 159,319 community residents, aged from 35 to 75, were enrolled from 2015 to 2020. Subjects born between 1 January 1955 and 31 December 1965, excluding those born between 1 January 1958 and 31 December 1958 and between 1 January 1962 and 31 December 1962, were selected. Finally, a total of 45,189 subjects were included in this study. This study was approved by the central ethics committee of China’s National Center for Cardiovascular Disease (Ethics approval No.:2014-574). All the participants signed the informed consent form.

### 2.2 Famine exposure definition

The Great Chinese Famine was a devastating disaster that occurred between the late 1950s and the early 1960s, resulting in millions of deaths nationwide. The severity of famine in each province was defined according to the estimated death rate (EDR), which was calculated as the change in mortality rate from the average level in 1956–1958 to the highest level over the period of 1959–1961 (9). The EDR cut-off value was set to 100% and regions with an EDR ≥100% were categorized as having experienced more severe famine, while the others were categorized as having experienced less severe famine. The EDR was 208.6 in Henan, ranking in the top five provinces most severely affected by famine between 1959 and 1961. Consistent with a previous Chinese famine study (6), the participants born between 1 January 1958 and 31 December 1958 and between 1 January 1962 and 31 December 1962 were excluded to reduce misclassification because the exact start and end dates of the famine were not clear. In most famine studies, the exposed group is defined as those born between 1959 and 1961, while the selection of control groups has been inconsistent. Considering the prolonged duration of the Great Chinese Famine and potential survivorship bias, only using post-famine birth cohorts as controls may underestimate the long-term effects. Therefore, in our study, the famine-exposed group was defined as participants born between 1 January 1959 and 31 December 1961, and the non-exposed group was defined as two populations combined, namely pre-famine births, including participants born between 1 January 1955 and 31 December 1957, and post-famine births, including those born between 1 January 1963 and 31 December 1965, to make the group age-balanced. This method was used in and recommended by previous studies on the Great Chinese Famine (10, 12–14).

### 2.3 CVD and 10-year CVD risk definitions

CVD was defined by answering an electronic questionnaire conducted by trained staff. Participants who answered in the affirmative to “Have you ever been diagnosed with an acute myocardial infarction (AMI) or stroke?” and provided the diagnosis year and/or indicated that they had undergone a percutaneous coronary intervention (PCI) and/or coronary artery bypass grafting (CABG) and the operation year it happened were classed as having CVD. The 10-year CVD risk was defined as ≥20% predicted by the Prediction for Atherosclerotic Cardiovascular Disease Risk in China (China-PAR) model, including age, sex, systolic blood pressure (SBP), currently smoking, diabetes, and total cholesterol (TC) (15).

### 2.4 Physical measurements and biochemical test

Each participant had a 5–10-min in-person interview with a trained staff member using a computer-delivered questionnaire. When the trained technicians measured their height and weight, each participant was asked to ensure they had an empty stomach and to wear light clothes without shoes or a cap. Their weight was measured on a scale that was accurate to the nearest 0.1 kg, and height was accurate to the nearest 0.1 cm. Body mass index (BMI) was calculated using the formula BMI = weight (kg)/height^2^(m^2^), and obesity was defined as BMI ≥ 28 kg/m^2^ (16). Blood pressure was measured on the right upper arm after 5 min of rest in a seated position using an electronic blood pressure monitor (OmronHEM-7430; Omron Corporation, Kyoto, Japan). SBP and diastolic blood pressure (DBP) were measured twice at 1-min intervals. If the difference between the two readings was 10 mmHg (1 mmHg = 0.133 kPa), a third blood pressure reading was measured and the mean value of the last two readings was calculated. Furthermore, 5 mL of fasting venous blood was collected in an ethylenediaminetetraacetic acid (EDTA) vacuum tube to test fasting blood glucose (FBG) using a BaiJie BK6-20md rapid blood glucose analyzer (QinLi Biotechnology Co., Xinbei, Taiwan). Total glyceride (TG), TC, high-density lipoprotein cholesterol (HDL-C) and low-density lipoprotein cholesterol (LDL-C) levels were tested using fingertip blood samples in a rapid lipid analyzer (CardioChek PA Analyzer; Polymer Technology Systems, Indianapolis, IN, USA). Hypertension was defined as self-reported anti-hypertensive drug use or SBP ≥140 mmHg and/or DBP ≥90 mmHg. Diabetes was defined as self-reported anti-diabetic drug use or FBG ≥7.0 mmol/L. Dyslipidemia was defined as self-reported lipid-lowering drugs use or TG ≥ 2.26 mmol/L or TC ≥ 6.22 mmol/L or HDL-C < 1.04 mmol/L or LDL-C ≥ 4.14 mmol/L.

### 2.5 Statistical methods and software

SAS Studio was used for the statistical analyses. Continuous variables are presented as mean ± standard deviation or median (lower quartile, upper quartile), and categorical variables are presented as *n* (%). Multivariate logistic regression models were used to explore the association between fetal exposure to famine and CVD in adulthood, with adjustments for age, sex, education, family’s annual income, currently smoking, drinking alcohol, BMI, anti-hypertensive drugs, anti-diabetic drugs, and lipid-lowering drugs. The odds ratios (OR) [95% confidence interval (CI)] estimated by the subgroup analyses stratified by sex and area were plotted in graphs using R version 4.3.2.

## 3 Results

### 3.1 Basic characteristics of the study participants

In total, 45,189 participants were included, of which 35,411 were in the non-exposed group and 9,778 were in the famine-exposed group. The median age was 57.0 years (54.0–60.0 years), and 17,399 were men (38.5%). Compared to the non-exposed group, the participants in the famine-exposed group had a higher prevalence of diabetes and had higher FBG and TC levels. The basic characteristics of the study participants are presented in Table 1.

**Table 1:** The basic characteristics of the participants in the study.

Variables	Total	Exposed	Non-exposed	*p*-value
N		9,778	35,411	
Sex				0.357
Male	17,399 (38.5%)	3,804 (38.9%)	13,595 (38.4%)	
Female	27,790 (61.5%)	5,974 (61.1%)	21,816 (61.6%)	
Age (years)	57.0 (54.0–60.0)	57.0 (56.0–59.0)	57.0 (53.0–61.0)	<0.001
Education				<0.001
High school and below	31,175 (69.0%)	6,059 (62.0%)	25,116 (70.9%)	
High school and above	14,014 (31.0%)	3,719 (38.0%)	10,295 (29.1%)	
Family’s annual income (RMB/year)				0.556
<5,000	39,119 (86.6%)	8,447 (86.4%)	30,672 (86.6%)	
≥5,000	6,070 (13.4%)	1,331 (13.6%)	4,739 (13.4%)	
Marriage status				0.722
Married	3,119 (6.9%)	667 (6.8%)	2,452 (6.9%)	
Other	42,070 (93.1%)	9,111 (93.2%)	32,959 (93.1%)	
Currently smoking				<0.001
No	36,420 (80.6%)	7,743 (79.2%)	28,677 (81.0%)	
Yes	8,769 (19.4%)	2,035 (20.8%)	6,734 (19.0%)	
Drink alcohol				0.689
No	41,057 (90.9%)	8,894 (91.0%)	32,163 (90.8%)	
Yes	4,132 (9.1%)	884 (9.0%)	3,248 (9.2%)	
Farm				<0.001
No	22,996 (50.9%)	5,164 (52.8%)	17,832 (50.4%)	
Yes	22,193 (49.1%)	4,614 (47.2%)	17,579 (49.6%)	
BMI (kg/m^2^)^a^				0.182
<18.5	430 (1.0%)	98 (1.0%)	332 (0.9%)	
18.5–23.9	14,885 (33.3%)	3,247 (33.6%)	11,638 (33.3%)	
24–27.9	20,033 (44.9%)	4,390 (45.4%)	15,643 (44.7%)	
≥28	9,299 (20.8%)	1,939 (20.0%)	7,360 (21.0%)	
Waist circumference	85.0 (79.0–91.0)	85.0 (79.0–91.0)	85.0 (79.0–91.0)	0.549
SBP	136.5 (125.0–149.5)	136.5 (125.0–149.0)	136.5 (125.0–149.5)	0.442
DBP	83.0 (76.0–90.0)	83.0 (76.0–90.0)	83.0 (76.0–90.0)	0.800
FBG	5.9 (5.3–6.8)	6.0 (5.4–6.8)	5.9 (5.3–6.7)	<0.001
TC	4.5 (3.8–5.2)	4.5 (3.8–5.2)	4.4 (3.8–5.1)	0.010
TG	1.3 (1.0–1.9)	1.3 (1.0–1.9)	1.3 (1.0–1.9)	0.167
HDL-C	1.3 (1.1–1.6)	1.3 (1.1–1.6)	1.3 (1.1–1.6)	0.309
LDL-C	2.4 (1.9–3.0)	2.4 (1.9–3.0)	2.4 (1.9–3.0)	0.004
Hypertension				0.708
No	20,766 (46.0%)	4,477 (45.8%)	16,289 (46.0%)	
Yes	24,423 (54.0%)	5,301 (54.2%)	19,122 (54.0%)	
Diabetes				<0.001
No	34,910 (77.3%)	7,353 (75.2%)	27,557 (77.8%)	
Yes	10,279 (22.7%)	2,425 (24.8%)	7,854 (22.2%)	
Dyslipidemia				0.355
No	39,600 (87.6%)	8,542 (87.4%)	31,058 (87.7%)	
Yes	5,589 (12.4%)	1,236 (12.6%)	4,353 (12.3%)	
Anti-diabetic drugs				0.013
No	42,581 (94.2%)	9,163 (93.7%)	33,418 (94.4%)	
Yes	2,608 (5.8%)	615 (6.3%)	1,993 (5.6%)	
Anti-hypertensive drugs				0.664
No	37,795 (83.6%)	8,164 (83.5%)	29,631 (83.7%)	
Yes	7,394 (16.4%)	1,614 (16.5%)	5,780 (16.3%)	
Lipid-lowering drugs				0.609
No	43,413 (96.1%)	9,385 (96.0%)	34,028 (96.1%)	
Yes	1,776 (3.9%)	393 (4.0%)	1,383 (3.9%)	

^a^ Data were missing.

### 3.2 Association between fetal exposure to famine and CVD in adulthood

Compared to the non-exposed group, exposure to famine in the fetal period increased the risk of total CVD (OR = 11.25, 95% CI: 1.14–1.38), coronary heart disease (CHD) (OR = 11.25, 95% CI: 1.05–1.47), stroke (OR = 11.22, 95% CI: 1.09–1.36), and 10-year CVD risk (OR = 11.22, 95% CI: 1.14–1.31) independent of age, sex, marriage, educational status, family’s annual income, currently smoking, drinking alcohol, BMI, and drug use, including anti-hypertensive drugs, anti-diabetic drugs, and lipid-lowering drugs. The associations between exposure to famine in early life and CVD risk in adulthood are shown in Table 2.

**Table 2:** Association between exposure to famine in the fetal period and CVD in adulthood.

Variables		CVD		MI		CHD		Stroke		10-year CVD risk	
		OR (95% CI)	*P*	OR (95% CI)	*P*	OR (95% CI)	*P*	OR (95% CI)	*P*	OR (95% CI)	*P*
Model 1^a^	Ref	1		1		1		1		1	
	Exposed group	1.19 (1.09–1.31)	<0.001	1.24 (1–1.53)	0.053	1.23 (1.05–1.45)	0.010	1.16 (1.04–1.28)	0.007	1.32 (1.23–1.41)	<0.001
Model 2^b^	Ref	1		1		1		1		1	
	Exposed group	1.21 (1.1–1.32)	<0.001	1.19 (0.96–1.48)	0.114	1.21 (1.03–1.42)	0.022	1.18 (1.07–1.32)	0.002	1.2 (1.12–1.28)	<0.001
Model 3^c^	Ref	1		1		1		1		1	
	Exposed group	1.21 (1.11–1.33)	<0.001	1.2 (0.97–1.48)	0.101	1.22 (1.04–1.43)	0.017	1.19 (1.07–1.32)	0.001	1.22 (1.14–1.31)	<0.001
Model 4^d^	Ref	1		1		1		1		1	
	Exposed group	1.25 (1.14–1.38)	<0.001	1.21 (0.98–1.51)	0.083	1.25 (1.05–1.47)	0.010	1.22 (1.09–1.36)	<0.001	1.22 (1.14–1.31)	<0.001

CVD, cardiovascular disease; MI, myocardial infarction; CHD, coronary heart disease.

^a^ Model was null.

^b^ Model 1 adjusted for age, sex, marriage, educational status, and family’s annual income.

^c^ Model 1 adjusted for age, sex, marriage, educational status, family’s annual income, currently smoking, and drinking alcohol.

^d^ Model 1 adjusted for age, sex, marriage, educational status, family’s annual income, currently smoking, drinking alcohol, BMI, anti-hypertensive drugs, anti-diabetic drugs, and lipid-lowering drugs.

### 3.3 Analyses stratified by sex and area

**Figure 1:**
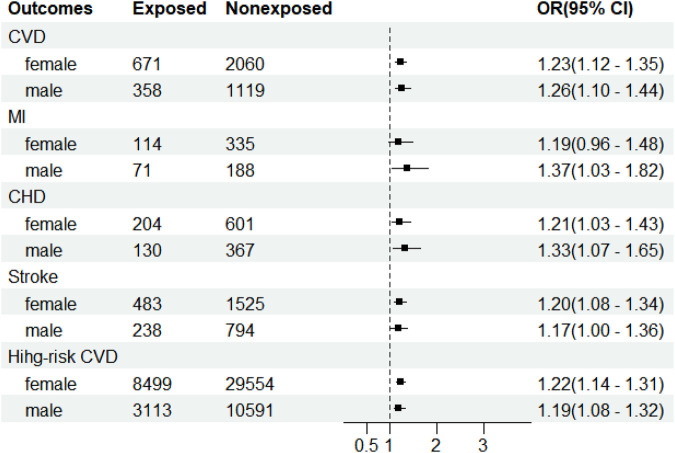
Adjusted odds ratios with 95% confidence interval for cardiovascular disease due to fetal exposure to the famine of 1959–1961 stratified by sex in the cross-sectional study conducted in Henan by the China PEACE Million Persons Project between 2015 and 2020. The ORs (95% CI) were estimated by the multivariate logistic regression models with adjustments for age, gender, marriage, educational status, family’s annual income, smoking, drinking alcohol, BMI, anti-hypertensive drugs, anti-diabetic drugs, and lipid-lowering drugs. CI, confidence interval; PEACE, Patient-Centered Evaluative Assessment of Cardiac Events; CVD, cardiovascular disease; MI, myocardial infarction; CHD, coronary heart disease.

The analysis stratified by sex showed, after adjustment for age, marriage, educational status, family’s annual income, currently smoking, drinking alcohol, BMI, and drug use, including anti-hypertensive drugs, anti-diabetic drugs, and lipid-lowering drugs, that after being exposed to famine during the fetal period, men had a higher risk than women for CVD (OR = 11.26, 95% CI: 1.10–1.44; OR = 11.23, 95% CI: 1.12–1.35, respectively) and CHD (OR = 11.33, 95% CI: 1.07–1.65; OR = 11.21, 95% CI: 1.03–1.43, respectively), while women had higher 10-year CVD risk than men (OR = 11.22, 95% CI: 1.14–1.31; OR = 11.19, 95% CI: 1.08–1.32, respectively). Male fetuses exposed to famine had increased risk of MI in adulthood (OR = 11.37, 95% CI: 1.03–1.82) and female fetuses exposed to famine had increased risk of stroke (OR = 11.20, 95% CI: 1.08–1.34), while no significant association was observed in for MI in women (OR = 11.19, 95% CI: 0.96–1.48, *p* = 0.117) or for stroke in men (OR = 11.17, 95% CI: 1.00–1.36, *p* = 0.05) (shown in Figure 1).

**Figure 2:**
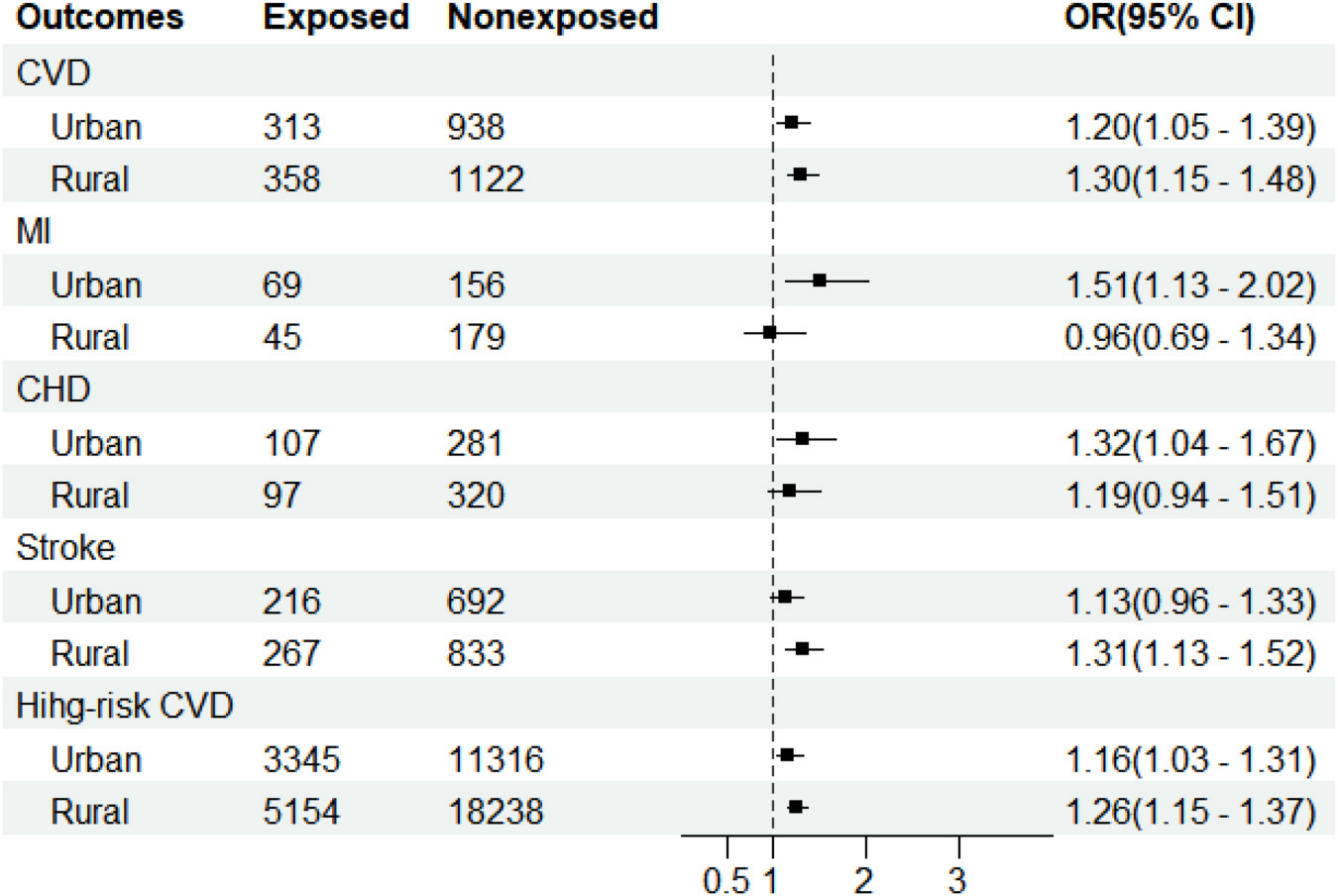
Adjusted odds ratios with 95% confidence interval for cardiovascular diseases due to fetal exposure to famine of 1959–1961 stratified by area in the cross-sectional study conducted in Henan by the China PEACE Million Persons Project between 2015 and 2020. The ORs (95% CI) were estimated by the multivariate logistic regression models with adjustments for age, gender, marriage, educational status, family’s annual income, smoking, drinking alcohol, BMI, anti-hypertensive drugs, anti-diabetic drugs, and lipid-lowering drugs. CI, confidence interval; PEACE, Patient-Centered Evaluative Assessment of Cardiac Events; CVD, cardiovascular disease; MI, myocardial infarction; CHD, coronary heart disease.

The association between exposure to famine during the fetal period and CVD was stronger in rural than in urban areas (OR = 11.30, 95% CI: 1.15–1.48; OR = 11.20, 95% CI: 1.05–1.39, respectively), as was the association between exposure to famine during the fetal period and 10-year CVD risk (OR = 11.26, 95% CI: 1.15–1.37; OR = 11.16, 95% CI: 1.03–1.31, respectively). In urban areas, exposure to famine increased the risk of an MI (OR = 11.51, 95% CI: 1.13–2.02) and CHD (OR = 11.32, 95% CI: 1.04–1.67) while in rural areas, exposure to famine increased the risk of stroke (OR = 11.31, 95% CI: 1.13–1.52) (shown in Figure 2).

## 4 Discussion

Based on a large population-based study, we observed that fetal exposure to famine increased the risk of CVD, CHD, stroke, and 10-year CVD risk in adulthood, independent of modifiable CVD risk factors. To our knowledge, this was the first study conducted in Henan Province to explore the association between undernutrition in the fetal period and CVD in adulthood.

Multiple studies based on the Developmental Origins of Health and Disease (DOHaD) hypothesis have examined the association between exposure to famine in early life and chronic diseases in adulthood, such as diabetes, obesity, hypertension, kidney stones, and CVD (6, 17–20), but the results have been inconsistent. In a study conducted in a Dutch famine cohort, a total of 7,845 female participants exposed at different ages (including 0–9, 10–17, ≥18 years) had increased CHD risk, with decreased stroke risk (21). A longitudinal study that recruited 259,657 community-dwelling adults aged 40 years or older across mainland China concluded that exposure to famine significantly increased the risk of total CVD, MI, stroke, and CHD (20). Among the 71,667 men and women who participated in the PEACE Million Persons Project in Guangdong, the exposed group had a higher risk of total CVD, CHD, AMI, heart failure (HF), and stroke in contrast to the non-exposed group (10). In two additional Dutch famine studies, however, no significant association was observed between famine exposure and CVD risk (22, 23). Similarly, Rotar et al. found that the famine caused by the siege of Leningrad had no direct effect on the prevalence of CVD (24). The discrepancy may be explained by the study design and the small sample size, and the different confounding factors adjusted for in these studies. In addition, the post food supply of Dutch famine was more rapid, and the duration of siege of Leningrad was short, which both could have caused an early postnatal catch-up growth effect (25). Another speculation was that the population developed a healthy lifestyle later, which weakened the malignant effect of the famine. Further study of the interaction effect of exposure to famine in early life and healthy habits on CVD is required.

We further analyzed the sex difference and found that the associations between fetal famine exposure and CVD, MI, and CHD risk in adulthood were all stronger in men than in women, which was inconsistent with previous studies (10, 26). The sex difference may be explained by male-sex preference in Chinese traditional culture, especially in Henan, which can result in women and girls being subjected to worse stressors than men and boys and the surviving female fetuses being more adaptable to adverse environments; thus, the effect was more pronounced in men (27). In addition, the biological fragility of male fetuses means that they are more vulnerable to adverse factors than their female counterparts (28) and the male survivors were more likely to live a rich material life than female survivors, thus they were at higher risk of adverse health effects, which is consistent with Barker’s hypothesis (29). Furthermore, we also found the risk of stroke due to fetal exposure to famine was higher in women, consistent with the China Kadoorie Biobank (CKB) study, a representative prospective study conducted in China to explore the risk factors of chronic disease (30).

Similarly, the association between fetal exposure to famine and CVD and 10-year CVD risk was stronger in rural areas than in urban areas, while fetal exposure to famine was associated with an increased risk of CHD and MI in urban areas but not in rural areas, which was consistent with the CKB study (30). The higher risk in rural areas may be attributed to famine severity, as rural residents suffered more severe famine than urban residents, which was supported by previous studies (6, 31). However, in this study, famine severity could not be defined owing to a lack of mortality data in cities. Furthermore, urban residents have more convenient and advanced healthcare, are more concerned about their physical condition, and have healthy habits. Thus, healthy habits could be argued to attenuate the effect of fetal famine exposure on CVD risk in adulthood, but this requires further study to verify.

The nature of the relationship between fetal famine exposure and CVD risk in adulthood has not yet been well elucidated, but several mechanisms have been discussed. First, stress in early life, through glucocorticoid signaling, has an inflammatory effect on the cardiovascular system and increases the risk of CVD (32). Second, after being exposed to famine in childhood, patients had a reduction in left ventricular outflow tract diameter, stroke volume, and cardiac output and markedly elevated peripheral resistance in adulthood, which suggests that childhood exposure to famine is associated with impaired cardiovascular structure and function and increased risk of CVD (33). Third, the adult survivors of malnutrition caused by famine in early life in the Dutch famine study had a preference for high-calorie food and had a higher prevalence of dyslipidemia (34), which then increased the occurrence of CVD (35). Fourth, findings from an epigenetic study proposed that early-life malnutrition could cause abnormal DNA methylation of genes, which persists throughout later life and results in structural changes in the cardiovascular system (36).

Several strengths of this study are worthy of mention. To our knowledge, this was the first study conducted in Henan Province to investigate whether exposure to famine in early life increases the risk of CVD in adulthood via a representative population-based study. This study provided evidence of an association between undernutrition in early life and CVD in adulthood. In addition, the large sample size guaranteed representation of the population and statistical significance. Importantly, the analytical approach used to recruit participants born before, during, and after the famine has been recognized as an effective and scientific way for famine studies to reduce misclassification and survivor bias to a large extent. Three limitations existed in this study. First, there was a lack of data on the famine severity in different counties across Henan Province; thus, the effect of famine severity on CVD risk could not be analyzed. Furthermore, diet and physical activity were not adjusted for because the data were not recorded in the initial screening questionnaire, and birth weight could not be controlled for, as the data could not be obtained. Finally, the study was cross-sectional, which does not provide evidence of causality between famine exposure and the incidence of CVD.

## 5 Conclusion

Our findings have significant public health implications. First, our results supported the DOHaD theory, providing scientific evidence for targeted policies and interventions aimed at CVD prevention at the population level, particularly in men and rural areas. In addition, our study indicated that low birth weight is associated with an elevated risk of chronic diseases in adulthood. This underscores the importance of maternal nutrition during childbearing age, even in an era where societal pressures often promote unnecessary weight loss. This observation reflects the broader socio-cultural influences on chronic disease risk. Furthermore, we emphasized that macro-level socioeconomic and political conditions can shape risk factors for CVD and other chronic diseases. Initiatives such as “Healthy Lifestyle for All” and “Chronic Disease Demonstration Areas” promote protective measures, such as healthy diets and smoke-free environments, mitigating population-level risk factors. These insights should inform evidence-based public health strategies and policies, fostering a multisectoral approach to curb the growing CVD epidemic and improve public health outcomes.

## Data Availability

The datasets presented in this article are not readily available because the authors need to grant permission to access the National Center for Cardiovascular Disease. Requests to access the datasets should be directed to jiangl@fwoxford.org.
